# 574. Pregnancy Outcomes in Patients with Burn Injury History: A Propensity-Matched Cohort Study

**DOI:** 10.1093/jbcr/irag033.314

**Published:** 2026-04-06

**Authors:** Laeticia P Evang, Pekam Njowo, Blancheneige M Beohon

**Affiliations:** University of Texas Medical Branch, Galveston, TX; University of Texas Medical Branch John Sealy School of Medicine, Galveston, TX; University of Texas Medical Branch, Galveston, TX

## Abstract

**Introduction:**

Severe burn injuries trigger systemic inflammation, metabolic stress, and long-term tissue remodeling, leading many survivors to worry about future reproductive risks. Evidence on pregnancy outcomes after burn injury is scarce, limiting clinicians' ability to provide reassurance. This study evaluated pregnancy outcomes among burn survivors compared with non-urn patients.

**Methods:**

We conducted a retrospective cohort study using the TriNetX Research Network, a federated electronic health record database. Pregnancies recorded between 2005 and 2023 were identified. Patients with a prior burn injury were compared with those without burn history. Propensity score matching (1:1) adjusted for age, race/ethnicity, hypertension, diabetes, anemia, and delivery history, resulting in two balanced cohorts of 62 227 patients each. Outcomes assessed at 3 and 6 months included fetal growth restriction, macrosomia, preterm delivery, gestational hypertension, preeclampsia, gestational diabetes, preterm premature rupture of membranes (PPROM), stillbirth, and abnormal placentation. Risk ratios (RR) with 95% confidence intervals (CI) were calculated; significance was defined as *p*<.05.

**Results:**

Unexpectedly, burn survivors demonstrated lower risks of several adverse outcomes compared with controls. At 3 months, reduced risks were observed for preeclampsia (RR 0.55, *p*<.0001), gestational hypertension (RR 0.63, *p*<.0001), gestational diabetes (RR 0.66, *p*<.0001), and preterm delivery (RR 0.67, *p*<.0001). These patterns persisted at 6 months. Stillbirth was rare and not significantly different between groups.

**Conclusions:**

Contrary to prevailing concerns, pregnancy after burn injury was not associated with increased risk. Instead, survivors experienced lower risks for several complications. These findings challenge assumptions and provide reassurance for patients and clinicians counseling on pregnancy after burn injury.

**Applicability of Research to Practice:**

This study provides evidence to guide counseling of burn survivors planning pregnancies. Clinicians can reassure patients that prior burn injury does not increase—and may even reduce—the risk of several adverse obstetric outcomes. These findings support more confident, patient-centered discussions, reducing unnecessary anxiety for survivors and improving shared decision-making in prenatal care.

**Funding for the study:**

Supported by the UTMB Institute for Translational Sciences (UL1 TR001439), funded by the National Center for Advancing Translational Sciences at the NIH.

